# Platelet-Rich Plasma for the Treatment of Ocular Surface Disease in Animals: A Systematic Review

**DOI:** 10.1155/vmi/9921619

**Published:** 2025-06-23

**Authors:** Daniel Uribe, Catalina López, Jorge U. Carmona

**Affiliations:** Department of Animal Health, Universidad de Caldas, Manizales, Colombia

**Keywords:** corneal ulcer, dog, keratoconjunctivitis sicca, ocular surface, platelet-rich plasma, rabbit, rat

## Abstract

**Background:** Platelet-rich plasma (PRP) is increasingly used to treat ocular surface diseases (OSDs) in animals, including corneal ulcers and keratoconjunctivitis sicca (KCS). However, existing studies lack rigorous evaluation of efficacy and safety. This systematic review assesses the quality, outcomes, and therapeutic benefits of PRP in veterinary ophthalmology.

**Methods:** A systematic review (January 2013–December 2023) of experimental and clinical studies in animals treated with PRP was conducted. From 126 records, 14 studies met inclusion criteria (rabbits, dogs). Outcomes included corneal healing, inflammation, and symptom relief.

**Results:** PRP showed significant benefits: improved corneal healing (12/14 studies), reduced edema/vascularization (9/14 studies), and decreased pain/inflammation (5/14). However, a high risk of bias (10/14 studies), small sample sizes (median *n* = 20), and inconsistent PRP protocols (i.e., unreported platelet concentrations in 7/14 studies) limit conclusions.

**Conclusions:** Despite promising results, PRP cannot yet be recommended as standard care due to methodological limitations. Future studies must standardize PRP preparation, such as centrifugation protocols, and platelet counts, prioritize randomized controlled trials (RCTs), and report adverse effects transparently.

## 1. Introduction

The mammalian ocular surface—including in humans and dogs—comprises three principal structures: the cornea, conjunctiva, and tear film. This specialized region contains three distinct epithelial types (corneal, limbal, and conjunctival), all nonkeratinized stratified squamous epithelia that exhibit unique regenerative properties following injury [1, 2]. The corneal epithelium exhibits rapid turnover, with complete renewal occurring every 5–7 days across mammalian species. This remarkable regenerative capacity stems from proliferative progenitor cells residing in the limbal stem cell niche at the sclerocorneal junction [3, 4].

The most common ocular surface diseases include corneal ulceration and dry eye syndrome, a condition diagnosed primarily in dogs and humans that results from a deficiency in the quantity or quality of tears [5–7]. Tears have a complex composition of water, salts, proteins, glycoproteins, carbohydrates, and lipids, which are fundamental substances for the nutrition of the ocular surface [8, 9]. Corneal ulceration consists of a loss of continuity in one or more corneal layers (epithelium, stroma, Descemet's, and endothelium), causing an associated inflammatory process, pain, and risk of perforation, depending on the depth of the lesion. Treatment includes antibiotics, lubricants, analgesics, and in more severe cases, microsurgery [10–12].

Dry eye syndrome is one of the most common OSDs in humans and dogs. Keratoconjunctivitis sicca (KCS) most commonly results from immune-mediated destruction of lacrimal gland tissue, wherein autoimmune responses target and compromise the gland's secretory function [13–15]. Current treatment regimens for KCS primarily involve topical immunomodulators (i.e., cyclosporine, tacrolimus) combined with lubricating agents. However, these therapies present significant limitations, including restricted availability in certain markets and the requirement for lifelong, frequent administration to maintain therapeutic efficacy [16–19]. Ocular surface diseases can cause loss of corneal transparency and cause visual impairment, a condition that affects quality of life by limiting patients' ability to perform daily activities. Therefore, in addition to the growing interest in regenerative therapies, therapeutic options for these diseases have emerged [20–22].

Regenerative medicine is a field of current interest in several medical specialties; it is based on the knowledge of the body's self-healing tools, such as undifferentiated cells and hemocomponents, which are capable of stimulating the proliferation and regeneration of tissues after damage [23–25]. The application of factors, either cellular or biochemical, that interfere with biological processes is one of the cornerstones of regenerative medicine. Among the hemocomponents, mainly autologous serum (AS) and platelet-rich plasma (PRP) have been studied due to their high content of growth factors (GFs), which are polypeptides synthesized as precursors that, in the face of tissue damage, stimulate cells to enter a cycle of growth and division that favors regeneration [26–29]. Unlike AS, PRP preparation requires anticoagulant-treated centrifugation to concentrate both plasma proteins and platelets, yielding significantly greater GF concentrations. These elevated bioactive components enhance mitogenic and chemotactic cellular responses, rendering PRP superior for tissue regeneration applications [26–30]. PRP is a hemocomponent highly concentrated in platelets, which are anucleated elements derived from the fragmentation of megakaryocytes. The internal granules of platelets are reservoirs of several biological mediators, such as GFs, cytokines, chemokines, adhesion proteins, protease inhibitors, and fibrinolytic factors, among others [31, 32].

There are several clinical studies evaluating the effects of PRP in both humans and animals, in areas such as surgery, dermatology, and musculoskeletal conditions [31, 32]. In human ophthalmology, PRP is considered a clinical tool with broad applicability due to its autologous properties and the absence of preservatives and stabilizers. In ocular surface diseases, there is an increased demand for GFs that affect corneoconjunctival maintenance and regeneration [26, 27, 29, 30, 33–37]. PRP has been used in ocular surface diseases such as dry eye, where it has been shown to improve patient signs [26, 34–37]. PRP has demonstrated therapeutic efficacy in corneal wound healing, particularly in ulcerative and perforating lesions, where its bioactive components significantly enhance epithelial cell migration rates [38]. It has also been used in humans for corneal transplant complications where it brought clinical improvement, as well as in chemical burns, and ophthalmic emergencies where it accelerated lesion resolution. It has also been used as a postoperative adjuvant in refractive and conjunctival surgery [39, 40].

Multiple clinical studies comparing PRP to conventional dry eye therapies have demonstrated superior improvement in both objective signs and subjective symptoms. Additionally, human trials have documented PRP's ability to significantly reduce key inflammatory mediators, including IL-1β, IL-6, and IL-8, suggesting an immunomodulatory mechanism of action [27, 41, 42]. Furthermore, few adverse effects associated with PRP use in ocular pathologies have been described; some cases of redness, pruritus, or infectious keratitis due to product contamination have been reported [30]. Furthermore, it is important to mention that several systematic reviews [43–45] have been conducted to evaluate the role of PRP and related hemocomponents in the treatment of human ocular surface disease. Collectively, these hemocomponents demonstrate clinically meaningful therapeutic effects, with PRP exhibiting particularly robust efficacy in promoting epithelial regeneration for moderate-to-severe corneal injuries [43–45].

While PRP has shown promise in human ophthalmology, its application in veterinary medicine remains poorly characterized, with no systematic reviews evaluating its efficacy for ocular surface diseases in animals. Given the translational potential of PRP therapy, rigorous assessment of its safety and effectiveness in veterinary patients is warranted. This systematic review aims to: (1) evaluate PRP's therapeutic outcomes in animal models and clinical cases, and (2) critically analyze preparation protocols to identify standardization gaps.

## 2. Materials and Methods

This systematic review did not require approval from the Institutional Board for Animal Experimentation because it is a study that does not involve direct experimentation on animals.

### 2.1. Systematic Review Procedure and PICO Strategy

An initial literature search was performed to identify keywords related to PRP and its clinical applications in veterinary ophthalmology. Then, based on the PRISMA (preferred reporting items for systematic reviews and meta-analyses) methodology [46–48], the PICO (population, intervention, comparison, outcome) keyword clustering strategy was used, with AND and OR connectors to link them. The term “appendices” was incorporated to refine the tissue-specific focus, while the Boolean operator “NOT” was applied to exclude studies involving human subjects, as preliminary searches yielded predominantly human-based research. The systematic search was then performed in several academic databases (PubMed, Science Direct, Scopus, SpringerLink, and the Nexus Academic Publishers), as follows: P: animal, cat, dog, horse, rabbit; I: PRP, platelet concentrate; C: not applicable. O: healing, regenerative; Appendix: cornea, eye, tear; NOT: human.

The selected databases (PubMed, ScienceDirect, Scopus, SpringerLink, Nexus Academic Publishers) were chosen to optimize literature coverage while balancing disciplinary focus and accessibility. PubMed provided comprehensive biomedical evidence despite human-study bias, while ScienceDirect and Scopus offered interdisciplinary breadth with full-text availability limitations. SpringerLink supplemented veterinary-specific content, and Nexus Academic Publishers ensured inclusion of open-access veterinary studies not indexed elsewhere. This strategy mitigated individual database limitations through cross-platform validation.

Using the above keywords and the AND, OR, and NOT connectors, the following search algorithm was developed: (animal OR cat OR dog OR horse OR rabbit) AND (“platelet-rich plasma”) AND (healing OR regenerative) AND (cornea OR eye OR tear) NOT (human). The search algorithm used for each database is described in Table 1.

### 2.2. Eligibility Criteria for Documents and Procedures for the Retrieval, Analysis, Screening, and Inclusion of Studies

Following finalization of the research question and search terminology, investigators conducted independent systematic searches across designated databases using their assigned keywords. Documents published in English, Spanish, and Portuguese were included in the study. The initially obtained registers were uploaded and managed in an online reference management application (EndNote Web, Clarivate, London, UK). Once the registers were uploaded, they were checked for duplicate documents. Then, each author evaluated the registers according to the inclusion criteria, considering the title, keywords, and abstract information. To ensure inter-rater reliability, any discrepancies in article selection were discussed and consensus was reached among the investigators through a review meeting. Inclusion criteria included clinical trials, experimental studies, case reports, and systematic reviews. In addition, the quality of experimental studies was assessed using the REFLECT statement [49], which evaluates the reporting quality of animal studies using a 22-item checklist, with each item scored as “yes”, “no” or “unclear”. A higher number of “yes” responses indicates better reporting quality. The quality of systematic reviews was assessed using the AMSTAR guideline [50], which evaluates the methodological rigor of systematic reviews using an 11-item scale. Items are scored as “yes,” “no,” “partial,” or “cannot answer,” and overall quality is rated as “high,” “moderate,” “low,” or “critically low” based on the presence of critical or noncritical methodological weaknesses.

On the other hand, exclusion criteria included articles whose title did not match the keywords; those performed on humans or tissues other than the ocular surface were discarded. Editorial and review article types were not included. A consensus review of the final article selection was conducted to ensure interinvestigator agreement on inclusion/exclusion criteria, thereby minimizing selection bias.

### 2.3. Assessment of the Studies According to Harrison and Alsousou Criteria

Selected studies were evaluated using standardized criteria designed to establish minimum reporting requirements for PRP research [51]. These 11-item criteria ensured objective assessment and enhanced reproducibility by mandating disclosure of essential methodological details. These criteria included 11 items, as follows: (1) The source of blood or platelets whether autologous or allogeneic. (2) The anticoagulant, volume, and age of blood used to prepare PRP. (3) The method used to prepare PRP. (4) The centrifugation conditions (g value, temperature, and time) used in the laboratory or within commercial PRP preparation devices. (5) If a commercial preparation device is used, then the make and batch numbers/expiry dates of disposables used to prepare the PRP should be included. (6) A full description of how the PRP is harvested (e.g., from buffy coats or PRP supernatants). (7) A measurement of the cellular content of the original whole blood and derived PRP including platelet count, white cell counts, and red blood cell counts and the methods used to count the cells. (8) The concentration factor and yield of platelets obtained. (9) A measure of quality of the PRP preparation (e.g. cellular content, platelet activation status, platelet specific proteins, and GF content. (10) Whether the PRP is activated before application either in vitro or in vivo, including the method used to activate the platelets before use and whether this converts plasma fibrinogen to form fibrin rich clots, and (11) the method and number of in vivo applications, the specific delivery sites, and volume of PRP administered [51].

### 2.4. Bias Risk Assessment

In addition to the REFLECT rating for experimental studies and the AMSTAR rating for systematic reviews, the quality of included studies was scored according to their risk of bias as serious (high), moderate (some concerns), and low, considering 5 domains (D) according to the Cochrane risk of bias criteria using the Robvis tool (https://www.riskofbias.info/welcome/robvis-visualization-tool) [52], as follows: D1: Bias arising from the randomization process. D2: Bias due to deviations from the intended intervention. D3: Bias due to missing outcome data. D4: Bias in the measurement of the outcome, and D5: Bias in the selection of the reported result.

To calculate the weighted overall risk of bias, each of the five bias domains was assessed and rated as low, moderate, or high. The individual domain scores were then combined to produce an overall risk of bias score. The Robvis tool aggregated these scores based on their importance in affecting study validity, with biases such as randomization (D1) or missing data (D3) weighted more heavily. The final weighted score was presented as a percentage or as a categorical classification (low, moderate, or high), reflecting the potential impact of the bias on the study's conclusions.

## 3. Results

**Figure 1:**
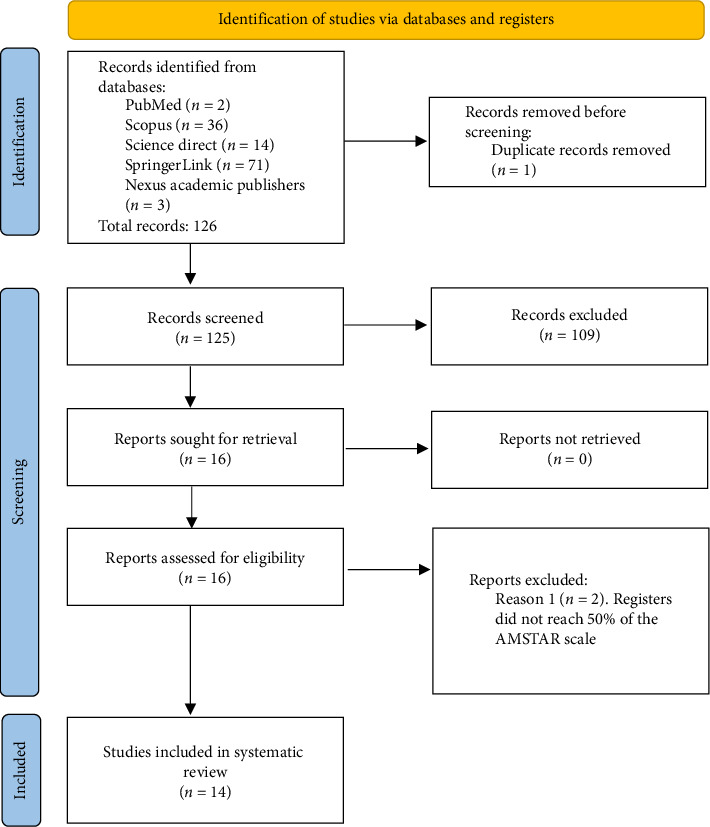
Flowchart of the study selection process according to the PRISMA.

The database search yielded 126 records for evaluation according to the inclusion and exclusion criteria. The search was conducted by individual investigators to reduce search bias due to differences in the databases consulted. Once the search was conducted, the inclusion and exclusion criteria were assessed and a total of 17 records were obtained, of which one record was found to be duplicated in the Science Direct and Scopus databases, and two registers did not exceed 50% of the AMSTAR score, resulting in 14 clinical trial-type articles for review (Figure 1).

### 3.1. General Study Analysis

The main animal model studied was the rabbit [53–59], most of which were of the New Zealand breed, except one article that did not specify the breed used [59]. The second most commonly used species was the dog [60–64], with no breed restriction. Two studies used the rat species as an animal model [65, 66], and one evaluated domestic cats [61].

Five of the 14 studies included in this systematic review were clinical trials conducted primarily in dogs, and one study investigated corneal ulcers in dogs and cats [61]. Four out of 14 studies, four evaluated the effect of PRP on corneal ulcers [60–63] and one in dogs with KCS [64]. Of note, the clinical studies were conducted in dogs and the experimental studies were conducted in rabbits [53–59] and rats [65, 66].

Two studies used only males [55, 66], three used only females [56, 57, 64], and the clinical trials used both males and females or did not specify the sex of the animal model used. The smallest sample size was found in the study by Acosta et al. [53], with 9 animals, and the largest sample size was found in the work by Gandolfi et al. [65], with 81 animals. One study [58] did not report the sample size used.

Among the clinical signs evaluated, pain, assessed by symptoms such as photophobia and blepharospasm, was included in three studies [53, 57, 62] and decreased in patients treated with PRP. Corneal edema and vascularization, as indicators of inflammation and opacity, were frequently evaluated [54, 57, 60, 62, 65], and decreased in patients treated with PRP in most articles. Ocular discharge was assessed in three studies [54, 57, 62], and uveitis, and conjunctival congestion in only one study [54], all of which were less common in patients treated with PRP.

Corneal healing, understood by concepts such as epithelialization, reconstruction, proliferation, and cell migration, were variables evaluated in most studies [53–63, 65, 66], in which it was found to be increased. Histologic findings related to the above, such as better stromal collagen arrangement and higher cell density, were evaluated in two studies [57, 66].

The studies that had KCS as a model disease evaluated tear production using the Schirmer test; one study found no changes in tear production [58] and the other found an increase [64].

Four studies found decreased corneal vascularization [54, 57, 63, 64] in eyes treated with PRP, while one study [59] found an increase in corneal vascularization. Two studies [55, 65] found that PRP produced a rapid hypervascularization, but also a rapid disappearance of vascularization, and two studies [60, 62] found no difference in vascularization. In addition, histopathologic findings were also a variable considered in some studies [54, 55, 57, 66], which found greater cell migration and better corneal remodeling in patients treated with PRP. Vatnikov et al. [64] evaluated corneoconjunctival inflammatory cellularity by cytology and found a decrease with PRP treatment.

Among the biomarkers evaluated in patients treated with PRP, two studies [55, 66] found an increase in alpha-smooth muscle actin (αSMA). This is a biomarker for the presence of myofibroblasts. Other biomarkers studied were matrix metalloproteinases (MMPs), which decreased in one study [61] and increased in another [65]. Some markers related to the oxidative cascade, such as malondialdehyde, which indicates the presence of free radicals, were found to decrease in patients treated with PRP. Catalase, an enzyme that converts hydrogen peroxide into water, has antioxidant activity and was found to increase [61]. In addition, Choi et al. [55] found a decrease in apoptosis markers such as bromodeoxyuridine and cytokines such as IL-1β.

Two studies evaluated KCS. One involved patients with this condition [64]. In the other, KCS was induced by applying 20 μL of 0.1% benzalkonium chloride topically three times daily for 3 weeks, followed by an increase to 0.2% for one more week [58]. The remaining studies evaluated ulcers or corneal lesions; four [58, 60–62] used animals with corneal lesions of various etiologies. In two studies [54, 57], the lesion was produced by burning with a 6-mm filter paper impregnated with sodium hydroxide on the cornea. In six studies [53, 55, 56, 59, 65, 66], a surgical corneal lesion was created. Some lesions were 3 mm in diameter [65, 66]. Another was 6 mm in diameter [59]. In one study [53], a surgical wound with a diameter of 10 mm was created. Two studies [55, 56] did not specify the diameter of the ulcer. In addition, only three studies [55, 56, 59] specified the depth of the lesion, two-thirds deep, epithelial only, and 0.25 mm, respectively.

Only one study [60] reported adverse effects, noting mild sensitivity in 5% of cases. No other included studies documented treatment complications. The remaining studies did not report any adverse effects associated with PRP therapy.  Table 2 provides a comprehensive synthesis of the 14 evaluated studies, detailing the investigative teams, animal models, specific ocular pathologies, treatment protocols (including administration routes and follow-up durations), primary outcome measures, and significant clinical and histopathological findings.

Table 3 provides a heatmap summarizing the effects of PRP and related products on clinical and histologic outcomes, along with overall study results. Across species—including rabbits, dogs, and rats—PRP demonstrated consistent benefits, significantly improving lesion area, corneal opacity, and vascularization. Notably, all canine studies reported highly favorable outcomes, suggesting particular efficacy in dogs. While most results were strongly positive, some variability was observed across measured parameters, supporting PRP's therapeutic potential despite minor inconsistencies.

A “highly positive” outcome was assigned to studies where PRP demonstrated high improvement in at least three out of four outcome variables (or all reported variables), such as lesion area, corneal opacity, vascularization, and cell migration. A “positive” outcome was assigned to studies where PRP showed moderate or high improvement in at least two outcome variables. These classifications aim to provide a standardized summary of PRP efficacy across the reviewed studies.

Species-specific trends have also been observed. In rabbits, PRP was highly effective, with most studies reporting consistently positive results. In dogs, PRP was consistently effective, with all studies showing highly positive results. In contrast, studies in rats were more limited, but the available data show highly positive results. Finally, a single study in cats also showed highly positive results with PRP treatment.

### 3.2. PRP Study Results According to Harrison and Alsousou Criteria

A summary with an analysis of the minimum reporting criteria in PRP studies is presented in Table 4. Of note, all studies failed to report critical data regarding the PRP used and its quality control. Seven studies did not report the platelet concentration in the evaluated PRP [53, 56, 57, 60, 61, 63, 64].

On the other hand, no study described the concentration of leukocytes in the PRP, nor the concentration of GFs or cytokines in the various PRPs evaluated.

Different blood centrifugation protocols were described to produce PRP. Most studies used double centrifugation protocols [54, 55, 57, 58, 62, 65, 66], with the first cycle being less intense than the second. Two studies [61, 63] used three centrifugation cycles and three studies [53, 56, 64] used a single centrifugation cycle, while two studies [59, 60] did not specify their technique in the scientific writing because they cited an author or technique as a reference. This variability in centrifugation protocols results in significant variability in the PRP produced, making it difficult to standardize the composition and quality across studies. Such inconsistency can directly affect the therapeutic outcomes of PRP treatments, as factors such as platelet concentration, leukocyte content, and mediator concentrations can vary dramatically depending on centrifugation settings.

On the other hand, three studies reported their centrifugation protocols in revolutions per minute (rpm), while one study used a human PRP protocol using blood from dogs; these facts make it impossible to reproduce these PRP procurement techniques.

On the other hand, blood for PRP procurement was obtained by several techniques. In the rat studies [65, 66] and in one rabbit study [57], blood was collected by intracardiac puncture. In other studies, blood was collected by jugular venipuncture [54, 60, 62]. The atrial marginal vein [53, 58] and the atrial central artery [56, 59] were also used for blood collection in rabbits. Another vein used for blood sampling in rabbits was the femoral vein [55], while the saphenous vein was also used in a dog study [64]. On the other hand, two studies did not specify the vein used for blood collection [61, 63].

Several key aspects of centrifugation protocols were inconsistently reported, including the revolutions per minute (rpm) used in the studies. Three studies [53, 55, 64] reported their centrifugation protocols in revolutions per minute (rpm), while one study [60] used a human PRP protocol using canine blood. These discrepancies in centrifugation parameters and reporting standards make it nearly impossible to replicate the PRP procurement techniques used in these studies, undermining reproducibility. Since centrifugation conditions are critical in determining the final composition of PRP, the lack of consistency and transparency in reporting these variables highlight the urgent need for standardized protocols to improve reproducibility and ensure reliable results in both clinical and preclinical studies.

### 3.3. Bias Risk Assessment

Based on the overall risk of bias analysis, 10 studies [53, 54, 56–59, 62–64, 66] had a high risk of bias, while 3 studies [55, 61, 65] had a moderate risk of bias and one study [60] had a low risk of bias. The studies with a high risk of bias generally had problems with sample size, methodological transparency, and quality control of the PRP. For example, Acosta et al. [53] had a very small sample size (9 animals), which could limit the statistical power and generalizability of the results and is considered a significant flaw. Furthermore, according to the criteria of Harrison and Alsousou [51], seven studies [53, 56, 57, 60, 61, 63, 64] did not report the platelet concentration of their PRP formulations, which affects the reproducibility of the studies and introduces a bias related to the quality of the PRP used in the treatments.

Another factor contributing to the high risk of bias is the lack of methodological consistency between studies. Centrifugation protocols for PRP preparation varied widely. They ranged from single to triple cycles. Some studies omitted important details such as rpm or the exact technique used. This variability can result in different concentrations of platelets, leukocytes, and GFs, leading to inconsistent therapeutic effects. To address these concerns, future research should establish and adhere to standardized centrifugation protocols, including specific details such as time and relative centrifugation forces, number of cycles, and other critical parameters that affect PRP quality. Researchers should also include platelet and leukocyte concentrations and other critical indicators of PRP quality in their reports to improve reproducibility and transparency. Clear and reproducible reporting of centrifugation techniques would help reduce variability between studies and increase the validity of results. The lack of clear protocols compromises reproducibility and contributes to potential performance bias.

**Figure 2:**
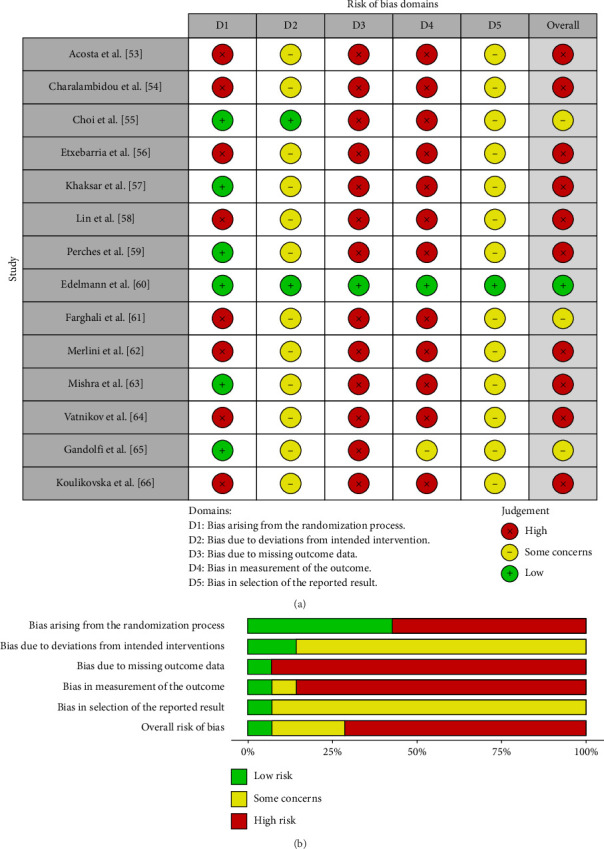
(a) Traffic light plots of the domain-level judgments for each study in the systematic review. Each study is color-coded (green, yellow, and red) to indicate the risk of bias across various domains. (b) Weighted bar plots showing the distribution of risk of bias ratings within each domain for all studies included in the review. The bars represent the proportion of studies classified as having low, unclear, or high risk of bias in each domain.

As shown in Figure 2(a), the traffic light plot summarizes the risk of bias for the 14 studies included in this systematic review and illustrates the overall risk of bias for each study. The majority of studies have a high risk of bias. The weighted overall risk of bias for all studies is 73%, as shown in Figure 2(b).

#### 3.3.1. Specific Design Flaws

Specific design flaws found in this systematic review were mainly related to sample size and sex selection. Lack of control groups and inconsistent quality control of PRP were also issues. The small sample sizes, such as in Acosta et al. [53] (9 animals), increase the risk of Type I error and low external validity. In addition, gender imbalance in some studies (i.e., using only male or female animals) contributes to gender bias and limits the generalizability of the results. Several studies lacked appropriate control groups—either placebo or alternative treatments. This limits the ability to determine whether observed effects were actually due to PRP or to other confounding factors. This lack of controls contributes to detection bias and performance bias. Most studies failed to report critical information about PRP quality. This includes GF concentrations, platelet and leukocyte levels, and the method of PRP preparation. As noted above, seven studies failed to report platelet concentrations, a critical aspect of reproducibility and therapeutic efficacy. This lack of standardization in PRP preparation increases the likelihood of biased results due to variability in the PRP product used. To mitigate these issues, researchers should take actionable steps such as reporting full details of PRP preparation protocols, including the use of standardized centrifugation techniques, precise platelet and leukocyte concentrations, and other quality control measures. In addition, studies should ensure larger and more representative sample sizes and include control groups to strengthen the evidence and reduce the risk of bias.

## 4. Discussion

This systematic review was conducted to evaluate the safety and therapeutic effect of PRP as a treatment for ocular surface disease in animals and to establish the quality process of the PRP evaluated in these research works. Therefore, the studies that met the inclusion criteria established in the methodology of this study included two major groups of research, divided into animal models of corneal damage (rabbits [53–59] and rats [65, 66]) and clinical trials in small animals, particularly dogs [60–63], with corneal ulcers and KCS [64].

In general, the outcome variables (i.e., corneal edema, corneal vascularization, lesion area, uveitis, corneal sensitivity, and corneal histopathology, among others) assessed in the studies included in this systematic review showed improvement in the animal models and in the pet patients treated with PRP compared to controls. The studies were consistent in improving corneal opacity and inflammation, reducing signs associated with ocular pain, and accelerating lesion repair, which may demonstrate that PRP and related hemocomponents are useful for the treatment of ocular surface diseases in animals, as demonstrated in rabbits [53–59], rats [65, 66], and dogs [60–64].

However, two major concerns arose after conducting this systematic review. One is related to the poor quality of the design of the studies included in this analysis (Figures 2(a) and 2(b)). The other is related to the inadequate description of the techniques for producing PRP together with the scarce information on the PRP used, such as cell (platelets, leukocytes, and leukocyte subpopulations) and mediator (GFs, cytokines, and chemokines) concentration [51].

Variability in PRP preparation protocols is one of the most critical issues identified in this review. Different centrifugation protocols, platelet concentrations, and blood collection techniques were used without clear rationale or standardization, likely contributing to inconsistent results between studies. This lack of consistency hampers cross-study comparisons and efficacy assessments, as variations in platelet/leukocyte counts and GF concentrations may lead to differing biological activities [67–70]. Future studies should investigate protocol modifications like optimizing cellular concentrations, adjusting centrifugation techniques, or exploring specific cell/mediator adjustments to improve outcomes.

Beyond PRP preparation variability, ethical and practical challenges exist in animal studies. Several studies used invasive blood collection methods like intracardiac puncture or repeated venipunctures [57, 65, 66], which raise welfare concerns and may compromise data validity through stress-induced effects [71]. Future research should prioritize less invasive techniques (e.g., peripheral vein access) while maintaining scientific rigor through careful study design.

This systematic review highlights five critical requirements for robust PRP studies: (1) adequately powered sample sizes (minimum *n* = 15 for preclinical, *n* = 40 for clinical trials) with balanced gender representation; (2) standardized PRP protocols specifying centrifugation parameters (× g force, time, temperature), platelet concentrations (× 10^6^/μL), and GF concentrations (PDGF-BB, TGF-β_1_ in pg/mL); (3) appropriate control groups (placebo/standard treatment); (4) randomized, blinded designs to minimize bias; and (5) complete reporting of methodology and outcomes.

These measures align with REFLECT [49] and CONSORT [72] guidelines to ensure reproducibility and clinical relevance. Future investigations should prioritize cross-species comparative studies to establish (1) interspecies variability in PRP responsiveness and (2) optimal, transferable preparation protocols. This dual approach would simultaneously broaden therapeutic applications while addressing the standardization challenges identified in both veterinary and human PRP research.

## 5. Conclusions

PRP demonstrates the therapeutic potential for OSD in animals, offering benefits such as accelerated corneal healing, reduced inflammation, and pain relief—particularly in refractory corneal ulcers and KCS. However, due to significant methodological limitations (i.e., inconsistent protocols, underreported PRP composition, and small sample sizes), PRP should not yet be considered standard care in veterinary ophthalmology. The current evidence supports its use only as an adjunct therapy for cases unresponsive to conventional treatments, with protocols validated.

To advance clinical translation, future studies must: (1) improve reporting rigor, (2) validate long-term efficacy and safety in multicenter trials, and (3) optimize species-specific protocols. Until such evidence is available, PRP should be employed cautiously within individualized, multimodal treatment plans.

## Figures and Tables

**Table 1:** Search algorithm structure for databases used in the study.

Database	Search algorithm structure
PubMed	(animal OR cat OR dog OR equine OR rabbit) AND (“platelet-rich plasma”) AND (regenerative) AND (eye) NOT (human)
Scopus	(animal OR cat OR dog OR equine OR rabbit) AND (platelet-rich plasma) AND (healing OR regenerative) AND (cornea OR eye OR tear) AND NOT (human)
Science direct	(animal OR cat OR dog OR equine OR rabbit) AND (“platelet-rich plasma”) AND (regenerative) AND (eye) NOT (human)
SpringerLink	(animal OR cat OR dog OR equine OR rabbit) AND (“platelet-rich plasma”) AND (regenerative) AND (eye) NOT (human)
Nexus academic publishers	(animal OR cat OR dog OR equine OR rabbit) AND (“platelet-rich plasma”) AND (regenerative) AND (eye) NOT (human)

**Table 2:** General summary of the 14 studies included in the systematic review.

Author	Animal model	*n*	Disease	Administration way	Evaluated treatments	Follow-up time	Outcome variables	Main findings
Acosta et al. [53]	Rabbit	9	Corneal ulcer	Topical	NaCl 0.9%, deproteinized calf's blood extract, and PRP	7 days	Lesion area	Increased re-epithelialization and improved signs for platelet-rich plasma (PRP)
Charalambidou et al. [54]	Rabbit	36	Corneal ulcer	Intrastromal	Untreated, doxycycline, PRP, and PRP + doxycycline	14 days	Corneal edema, corneal vascularization, lesion area, uveitis, corneal sensitivity, histopathology	Rapid healing, less vascularization, and edema for PRP
Choi et al. [55]	Rabbit	33	Corneal ulcer	Gel covered with pig intestinal submucosa (PIS)	Untreated, PIS, PRP, platelet-rich fibrin (PRF)	8 week	Corneal vascularization, corneal opacity, histopathology, αSMA.	Faster onset and disappearance of vascularization for PRP, faster transparency for PRP, lower neutrophil population for PRP alone than untreated wound.
Etxebarria et al. [56]	Rabbit	14	Corneal ulcer	Topical	Various concentrations of PRGF, fetal bovine serum	4 week	Cell proliferation in vitro, lesion area, histopathology	Similar stimulation of cell migration, marked lesion repair without significant vascularization
Khaksar et al. [57]	Rabbit	20	Corneal ulcer	Subconjunctival	Untreated, acetylcysteine (AC), PRP, PRP + AC	3 week	Area of lesion, corneal opacity, blepharospasm, corneal vascularization, eye discharge	Increased repair speed, reduced opacity, corneal vascularization, and blepharospasm for PRP
Lin et al. [58]	Rabbit		Keratoconjunctivitis sicca	Topical	PRP, sacchachitin, PRP + sacchachitin	5 days	Lesion area, histopathology, cell migration, and cell viability	Faster healing for PRP, better effect on cell migration PRP + sacchachitin
Perches et al. [59]	Rabbit	45	Corneal ulcer	Topical	PRP, platelet-poor plasma (PPP)	4 week	Area of injury, vascularization, and corneal edema, metalloproteinases	Increased vascularization for PRP, increased epithelial cells at the lesion edge for PRP, increased expression of metalloproteinases for PRP.
Edelmann et al. [60]	Dog	40	Corneal ulcer	Topical	PRP, artificial tears	4 week	Corneal vascularization, corneal fibrosis	Increased vascularization, fibrosis, and % healing for PRP
Farghali et al. [61]	Cat, dog	28	Corneal ulcer	Subconjunctival	PRP	6 month	Antioxidant capacity, malondialdehyde, catalase, metalloproteinase 2 and 9. Corneal opacity, vascularization, lesion area, PDGF-BB concentration	Higher antioxidant capacity and lower concentration of metalloproteinases for PRP. Increased concentration of PDGF-BB for PRP, lower corneal opacity for PRP
Merlini et al. [62]	Dog	19	Corneal ulcer	Topical plus clot covered with nictitating membrane	PRP, PRP clot	4 week	Blepharospasm, photophobia, conjunctival hyperemia, chemosis, discharge, opacity, and corneal vascularization	Improvement in all variables for PRP
Mishra et al. [63]	Dog	30	Corneal ulcer	Topical	PRP, fibrin-rich platelets and leucocytes (L-PRF)	4 week	Lesion area, corneal opacity, and corneal vascularity	L-PRF heals faster than PRP, both similar in vascularization and corneal opacity
Vatnikov et al. [64]	Dog	20	Keratoconjunctivitis sicca	Subconjunctival	PRP + cyclosporine + ciprofloxacin (CP) + artificial tear, NaCl + cyclosporine + CP + artificial tear	8 week	Schirmer's test, corneal opacity, corneal cytology	Greater and faster increase in tear production for PRP, decrease in opacity, and more marked cellularity for PRP
Gandolfi et al. [65]	Rat	81	Corneal ulcer	Topical	Untreated, PRP, and heated PRP	5 days	Corneal opacity, vascularization, lesion area, PDGF-BB concentration, PDGF-BB concentration	Increased PDGF-BB concentration for PRP, decreased corneal opacity for PRP
Koulikovska et al. [66]	Rat	40	Corneal ulcer	Topical	NaCl, PRP	5 days	Bromodeoxyuridine, αSMA and IL-1β.	Decreased apoptosis for PRP, no change in IL-1β expression

*Note:* C, centrifugation episode; PDGF-BB, platelet-derived growth factor; IL-1β, interleukin-1 beta.

Abbreviations: αSMA = alpha-smooth muscle actin; PRGF = plasma-rich in growth factors.

**Table 3:** Heatmap showing the overall outcome of platelet-rich plasma treatment on clinical and histology parameters across studies.

Author	Species	Clinical or histology parameter				
		Lesion area	Corneal opacity	Vascularization	Cell migration	Overall outcome^∗^
Acosta et al. [53]	Rabbit	High improvement	Moderate improvement	High improvement	NR	Positive
Charalambidou et al. [54]	Rabbit	High improvement	High improvement	High improvement	NR	Highly positive
Choi et al. [55]	Rabbit	High improvement	High improvement	High improvement	NR	Highly positive
Etxebarria et al. [56]	Rabbit	Moderate improvement	Moderate improvement	Moderate improvement	High improvement	Positive
Khaksar et al. [57]	Rabbit	High improvement	High improvement	High improvement	NR	Highly positive
Lin et al. [58]	Rabbit	High improvement	NR	NR	High improvement	Positive
Perches et al. [59]	Rabbit	Moderate improvement	Moderate improvement	High improvement	NR	Positive
Edelmann et al. [60]	Dog	Moderate improvement	Moderate improvement	High improvement	NR	Positive
Farghali et al. [61]	Cat, dog	High improvement	High improvement	High improvement	NR	Highly positive
Merlini et al. [62]	Dog	High improvement	High improvement	High improvement	NR	Highly positive
Mishra et al. [63]	Dog	High improvement	High improvement	High improvement	NR	Highly positive
Vatnikov et al. [64]	Dog	High improvement	High improvement	High improvement	NR	Highly positive
Gandolfi et al. [65]	Rat	High improvement	High improvement	High improvement	NR	Highly positive
Koulikovska et al. [66]	Rat	High improvement	NR	NR	NR	Positive

^∗^Highly Positive: studies where PRP showed high improvement in at least three out of four outcome variables (or all reported variables). Positive: studies where PRP showed moderate or high improvement in at least two outcome variables. Neutral/No Change: studies where PRP showed no significant improvement (not applicable in this dataset). No Data: studies where insufficient data were reported to determine overall outcome (not applicable in this dataset). NR: data not reported.

**Table 4:** Characteristics of PRP used in the studies according to Harrison and Alsousou criteria.

Author	Characteristic (C)^∗^										
	C1	C2	C3	C4	C5	C6	C7	C8	C9	C10	C11
Acosta et al. [53]	AUT	SC, 3.2%, 2.5 mL	Manual	1000 rpm/5 min	NA	BC	NR	NR	NR	NR	Corneal drops

Charalambidou et al. [54]	ALL	ACD, 8.7 mL	Manual	1^st^ C: 72 g/15 min 2^nd^ C: 1006 g/5 min 4°C	NA	BC	642.3 × 10^3^ PLT/μL	670%	4950.4 × 10^3^ PLT/μL	NR	Intrastromal

Choi et al. [55]	NR	SC, 3.8%, 15 mL	Manual	1^st^ C: 1600 rpm/12 min 2^nd^ C: 2000 rpm/10 min	NA	SN	433.6 × 10^3^ PLT/μL	NR	1166.3 × 10^3^ PLT/μL	5% CaCl_2_	Intrastromal

Etxebarria et al. [56]	NR	SC, 3.8%, 7.5 mL	Manual	658 g/4 min	NA	SN	NR	NR	NR	CaCl_2_	Corneal drops

Khaksar et al. [57]	NR	CD, 10.3%	Manual	1^st^ C: 72 g/15 min 2^nd^ C: 1006 g/5 min	NA	SN	NR	NR	NR	NR	Subconjunctival

Lin et al. [58]	NR	ACD-A, 9 mL	Manual	1^st^ C: 720 g/15 min 2^nd^ C: 2650 g/15 min	NA	BC	NR	NR	642.3 × 10^3^ PLT/μL	Low-molecular-weight chitosan (135 μg)	Corneal drops

Perches et al. [59]	AUT	NR	NR	22°C	NA	NR	362.2 × 10^3^ PLT/μL	5.5 ×	1952.3 × 10^3^ PLT/μL	NR	Corneal drops

Edelmann et al. [60]	AUT	SC, 32 mL	Semi-automated	1^st^ C, 14 min	NR	BC	NR	NR	NR	NR	Corneal drops

Farghali et al. [61]	AUT	SC, 3.8%.	Manual	1^st^ C: 250 g/10 min 2^nd^ C: 2000 g/10 min 3^rd^ C: 3000 g/20 min	NA	BC	NR	NR	NR	CaCl_2_	Subconjunctival

Merlini et al. [62]	AUT	SC, 3.2%, 20 mL	Manual	1^st^ C: 220 g/10 min 2^nd^ C: 600 g/10 min	NA	BC	NR	3.96 ×	1416.3 × 10^3^ PLT/μL	10% CaCl_2_	Corneal drops

Mishra et al. [63]	AUT	ACD, 3.2%, 22.5 mL	Manual	1^st^ C: 200 g/10 min 2^nd^ C: 400 g/5 min 3^rd^ C: 600 g/5 min	NA	BC	NR	NR	NR	NR	Corneal drops

Vatnikov et al. [64]	AUT	NR	Manual	1550 rpm/10 min	NA	BC	NR	NR	NR	NR	Subconjunctival

Gandolfi et al. [65]	ALL	4.5 mL	Manual	1^st^ C: 220 g/10 min 2^nd^ C: 660 g/10 min	NA	BC	NR	4.9 ×	2525 × 10^3^ PLT/μL	NR	Subconjunctival

Koulikovska et al. [66]	ALL	CPD (0.15 mg/mL), 10 mL	Manual	1^st^ C: 220 g/20 min 2^nd^ C: 480 g/20 min	NA	SN	NR	NR	1800 × 10^3^ PLT/μL	NR	Corneal drops

∗C1: the source of blood or platelets whether autologous or allogeneic. C2: the anticoagulant, volume, and age of blood used to prepare PRP. C3: the method used to prepare PRP. C4: the centrifugation conditions (g value, temperature, and time) used in the laboratory or within commercial PRP preparation devices. C5: if a commercial preparation device is used, then the making and batch numbers/expiry dates of disposables used to prepare the PRP should be included. C6: a full description of how the PRP is harvested (e.g., from buffy coats or PRP supernatants. C7: a measurement of the cellular content of the original whole blood and derived PRP including platelet count, white cell counts, and red blood cell counts and the methods used to count the cells. C8: the concentration factor and yield of platelets obtained. C9: a measure of quality of the PRP preparation (e.g., cellular content, platelet activation status, platelet specific proteins, and growth factor content. C10: whether the PRP is activated prior to application either in vitro or in vivo, including the method used to activate the platelets before use and whether this converts plasma fibrinogen to form fibrin-rich clots. C11: the method and number of in vivo applications, the specific delivery sites, and volume of PRP administered. ACD, acid citrate dextrose; ALL, allogeneic; AUT, autologous; BC, buffy coat; C, centrifugation; CPD, citrate phosphate dextrose; NA: not applicable; NR, nonreported; SC, sodium citrate; SN, supernatant.

## Data Availability

Data sharing is not applicable to this article as no new data were created or analyzed in this study.
